# Resolvin E1 attenuates murine psoriatic dermatitis

**DOI:** 10.1038/s41598-018-30373-1

**Published:** 2018-08-08

**Authors:** Yu Sawada, Tetsuya Honda, Satoshi Nakamizo, Atsushi Otsuka, Narihito Ogawa, Yuichi Kobayashi, Motonobu Nakamura, Kenji Kabashima

**Affiliations:** 1 Department of Dermatology, Kyoto University Graduate School of Medicine, Kyoto, Japan; 2 Department of Dermatology, University of Occupational and Environmental Health, Kitakyushu, Japan; 3 Department of Biomolecular Engineering, Graduate School of Bioscience and Biotechnology, Tokyo Institute of Technology, Tokyo, Japan; 4 Singapore Immunology Network (SIgN) and Institute of Medical Biology, Agency for Science, Technology and Research (A*STAR), Biopolis, Singapore

## Abstract

The potential of omega-3 poly-unsaturated fatty acids (PUFAs) as a therapeutic target for psoriasis, a chronic inflammatory skin disease of IL-23/IL-17 axis, is a long-disputed question, since various epidemiological studies have suggested the association between high-intake of omega-3 PUFAs and the reduced frequency and severity of psoriasis. However, their actual significance and the molecular mechanisms remain largely unknown. To address these issues, we focused on resolvin E1 (RvE1), an omega-3 PUFAs-derived metabolite, and examined its effects on psoriatic dermatitis, using an imiquimod-induced mouse psoriasis model. RvE1 potently suppressed the inflammatory cell infiltration and epidermal hyperplasia in the psoriatic skin. RvE1 decreased the mRNA expression of IL-23 in the skin. Consistently, RvE1 inhibited IL-23 production by dendritic cells (DCs) *in vitro*. Furthermore, RvE1 exerted inhibitory effects on migration of cutaneous DCs and γδ T cells, a major IL-17-producing cell population in mouse, both *in vivo* and *in vitro*. These suppressive effects of RvE1 were mediated by its antagonistic function on BLT1, a receptor of leukotriene B4, and were also observed in human DCs, Th17 and Tc17 cells. Our results indicate a novel mechanism of omega-3 PUFA-mediated amelioration of psoriasis, and suggest a potential of RvE1 as a therapeutic target for psoriasis.

## Introduction

Psoriasis is a common, chronic inflammatory skin disease characterized by scaly erythematous plaques with marked epidermal hyperplasia^1^. Although its etiology still remains unknown, accumulating evidence indicates that cytokines including tumor necrosis factor α (TNF-α), IL-23 and IL-17 play pivotal roles in its development^2^. In the psoriatic lesions, it is considered that inflammatory dermal dendritic cells (DCs) produce IL-23, which facilitates the production of IL-17 from T helper 17 cell (Th17) or T cytotoxic 17 cell (Tc17) in the skin, leading to epithelial hyperplasia and inflammatory cell infiltration^3^. Indeed, biologics targeting these cytokines show potent efficacy on psoriasis^2^. However, these therapies have several unsolved issues, one of which is a possible increased risk of infectious diseases^4^. Therefore, novel types of anti-psoriasis drugs are still strongly required.

It has been reported that the development of psoriasis is affected by various daily life style-related factors, including diets^5^. Among the nutritional components of diets, fatty acids, especially omega-3 polyunsaturated fatty acids (PUFAs), have been suspected to be involved in the regulation of psoriasis. Omega-3 PUFAs are essential fatty acids that must be consumed as part of the daily diet. Epidemiological studies have demonstrated that individuals with high intake of omega-3 PUFA-containing foods, such as fish oils and nuts, have reduced frequency and severity of psoriasis^6^. In addition, several studies have demonstrated that administration of omega-3 PUFAs ameliorates the degree of skin inflammation in patients with psoriasis^7^. Thus, the antipsoriatic effects of omega-3 PUFAs have long been suspected, however, their contributions and mechanisms remain largely unknown.

Omega-3 PUFAs produce various metabolites with anti-inflammatory and proresolving properties^7–9^. Resolvin E1 (RvE1) is one such metabolite, and is known to have two receptors, BLT1 and ChemR23^10^. RvE1 plays an antagonistic effect on BLT1, a leukotriene B4 (LTB4) receptor, but exerts an agonistic effect on ChemR23. We and others have previously reported the anti-inflammatory roles of RvE1 in several inflammatory disease models, including colitis^11^, asthma^12^ and contact hypersensitivity (CHS)^13^. However, whether RvE1 possesses antipsoriatic effects has not been evaluated yet.

To understand the underlying regulatory mechanisms of omega-3 PUFAs in psoriasis, we focused on RvE1 and investigated its function in psoriatic dermatitis, using an imiquimod (IMQ)-induced murine psoriasis model^14^. Administration of RvE1 reduced the development of the psoriatic dermatitis. RvE1 inhibited the IL-23 production by DCs both *in vivo* and *in vitro*. RvE1 also impaired the migration of cutaneous DCs and γδT cells to the draining lymph nodes (dLNs), an essential step for the full development of this psoriasis model. RvE1 exerted these inhibitory effects possibly through blockade of LTB4-BLT1 signaling. Our results show the novel antipsoriatic mechanisms by omega-3 PUFAs metabolites, and may propose a potential of RvE1 as a candidate for novel antipsoriatic drug.

## Results

### RvE1 impairs IMQ-induced psoriatic dermatitis

**Figure 1:**
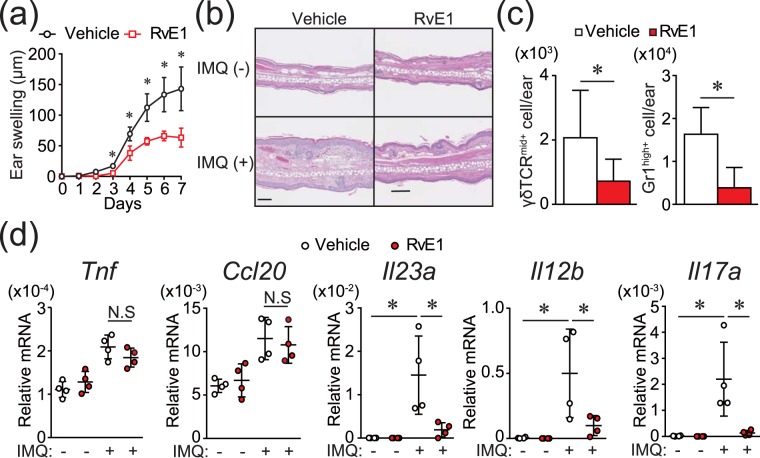
RvE1 inhibits IMQ-induced psoriatic skin inflammation. (**a**) Ear thickness change after topical IMQ application in mice treated daily with or without RvE1 (n = 4 per group). (**b**) Hematoxylin and eosin-stained sections of ear skin at day 7.  (Original magnification ×400. Scale bar, 100 μm). (**c**) The number of γδTCR^mid+^ γδ T cells, and Gr1^high+^ neutrophils in the skin at day 7. (**d**) The mRNA expressions of *Tnf* and *Ccl20* in the skin at day 1, and *Il23a*, *Il12b*, and *Il17a* in the skin at day 2. Results are expressed as the mean ± standard error of the mean (SD). All *p*-values were obtained by Student’s *t-*test: *p < 0.05. N.S indicates no significant difference. Data are representative of at least three independent experiments.

To examine the effect of RvE1 on the psoriatic dermatitis, we used an IMQ-induced mouse psoriasis model, a frequently used animal model of psoriasis^14^. Daily intravenous administration of RvE1 caused a significant decrease in the ear swelling and epidermal hyperplasia induced by IMQ (Fig. 1a,b). The numbers of dermal γδ T cells, a major IL-17-producing cell population in mice, and neutrophils in the inflamed skin were also significantly reduced in the RvE1-treated group (Fig. 1c).

The development of IMQ-induced psoriatic dermatitis is critically dependent on IL-23/IL-17 axis^14^. TNF-α or CCL20 also play significant roles for the induction of inflammatory cytokines or recruitment of γδ T cells in the skin^15,16^. To further evaluate the antipsoriatic effect of RvE1, we next examined the fluctuation of the cytokine expression in the skin under RvE1 treatment. Although no significant reduction was observed in *Tnf* and *Ccl20* mRNA expressions, the expressions of *Il23a*, *Il12b*, and *Il17a*, were downregulated by RvE1 (Fig. 1d).

Together, these results indicate the antipsoriatic effects of RvE1 in the IMQ-induced mouse psoriasis model.

### Inhibitory effects of RvE1 on IL-23 production in DCs, and involvement of BLT1 on the antipsoriatic effects of RvE1

**Figure 2:**
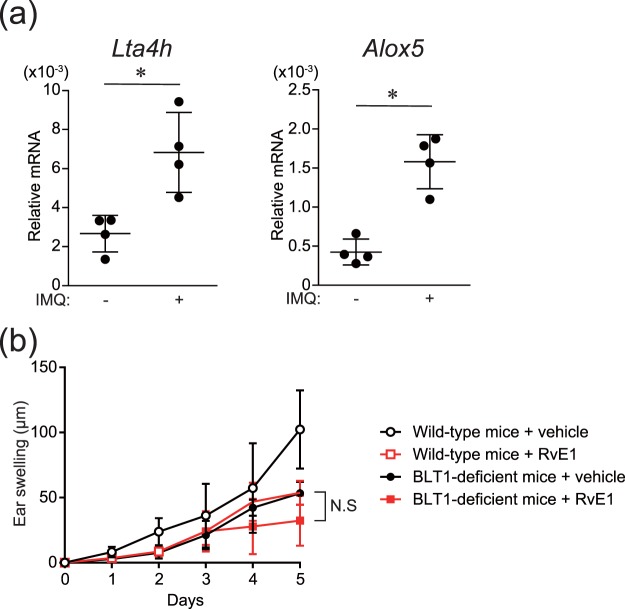
The mRNA expressions of LTB4 synthases in the skin and the effects of RvE1 on BLT1-deficient mice in the IMQ-induced psoriasis model. (**a**) The relative amount of *Lta4h* and *Alox5* was measured by mRNA extracted from ear skin before and 3 h after topical IMQ application. (**b**) Ear thickness change was measured for five consecutive days after topical IMQ application in wild-type and BLT1-deficient mice treated daily with either vehicle or RvE1 (n = 3 per group). N.S indicates no significant difference. Data are representative of two independent experiments.

We have previously reported that RvE1 exerts anti-inflammatory roles in CHS through the blockade of BLT1, an LTB4 receptor^13^. In IMQ-treated skin, a significant increase in the expression of LTB4 synthases, such as *Lta4h* and *Alox5*, was observed (Fig. 2a). In addition, BLT1-deficient mice in the psoriasis model exhibited attenuated ear swelling responses similar to RvE1-treated wild-type mice, and RvE1 did not exert further inhibitory effects on BLT1-deficient mice (Fig. 2b). These results suggest that RvE1 exerts antipsoriatic effects through the blockade of BLT1, thus, we further investigated the regulatory mechanisms of RvE1 focusing on LTB4-BLT1 signaling.

**Figure 3:**
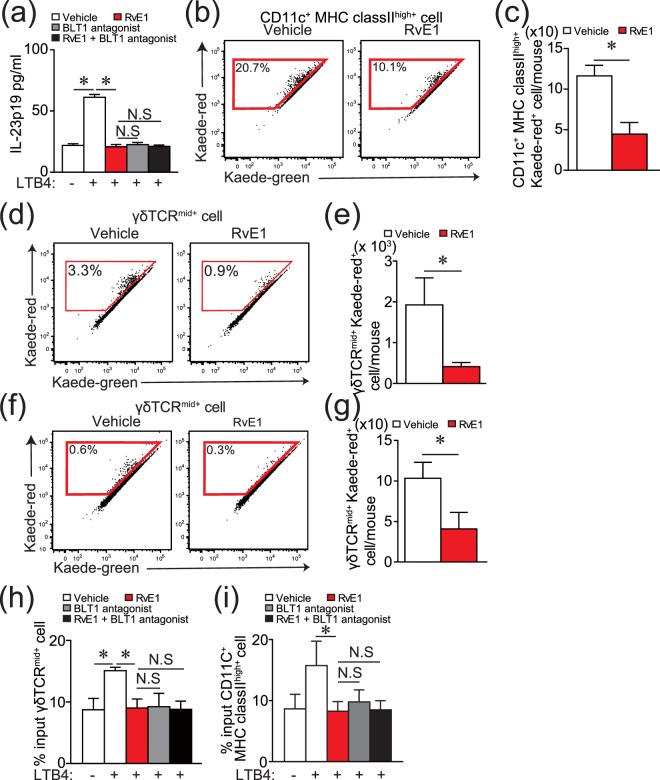
RvE1 inhibits the production of IL-23 by DCs and migration of DCs and γδ T cells. (**a**) The effects of LTB4 (10 nM), RvE1(100 nM), and a BLT1 antagonist (U-75302) (10 nM) on IL-23p19 production by bone marrow-derived DCs. The amount of IL-23p19 in culture supernatant was measured by ELISA. (**b**,**c**) A representative FACS plot of Kaede red^+^ cells (**b**) gated on DCs in Kaede transgenic mice, and the number (**c**) in dLNs of Kaede transgenic mice after IMQ application with or without RvE1 treatment. (**d**,**e**) Representative FACS plots of Kaede red^+^ cells (**d**) gated on γδTCR^mid+^ cells and the number (**e**) in dLNs after IMQ application with or without RvE1 treatment. (f and g) Representative FACS plots of Kaede red^+^ cells (**f**) gated on γδTCR^mid+^ cells, and the number (**g**) in dLNs of Kaede transgenic mice treated with or without RvE1 treatment in the steady state. (**h**,**i**) Transwell migration assay for γδTCR^mid+^ cells (**h**) and BMDCs (**i**). Thirty minutes after RvE1 (100 nM), a BLT1 antagonist (10 nM) or vehicle treatment, cells were tested for transmigration for 3 h (γδTCR^mid+^ cells) or 5 h (BMDCs) in the presence of vehicle or LTB4 (10 nM) in the lower chamber. Results are expressed as the mean ± SD. All *p*-values were obtained by Student’s *t-*test: *p < 0.05. N.S indicates no significant difference. Data are representative of at least three independent experiments.

Because RvE1 inhibited the expression of *Il23a* in the psoriatic lesions induced by IMQ (Fig. 1d), we next examined the effects of LTB4 and RvE1 on IL-23p19 production by DCs, using bone marrow-derived DCs. LTB4 significantly upregulated IL-23p19 production, which was inhibited by RvE1 to a similar extent as that of a BLT1 antagonist (Fig. 3a). Furthermore, RvE1 did not enhance the inhibitory effects of the BLT1 antagonist (Fig. 3a). On the other hand, although the expression of *Il17a* in the skin was inhibited by RvE1 treatment (Fig. 1d), no inhibitory effects of RvE1 were observed in IL-17A production by γδ T cells (Supplementary Fig. 1). These results suggest that part of the antipsoriatic effects by RvE1 is through the inhibition of IL-23 production by DCs *via* the blockade of LTB4-BLT1 signaling.

### Suppression of DC and γδ T cell migration by RvE1

In the IMQ-induced psoriasis model, cutaneous DCs migrate from skin to the dLNs and induce the proliferation of IL-17 producing-γδ T cells in dLNs^17^. In addition, it is reported that cutaneous IL-17 producing-γδ T cells also migrate to the dLNs, proliferate there, and recirculate to the psoriatic lesions to amplify the inflammation^18^. Therefore, we next investigated the effect of RvE1 on the migration ability of DCs and cutaneous γδ T cells from skin to the dLNs under the psoriatic inflammation. To analyze this, we applied a photolabeling system using Kaede transgenic mice. Kaede is a photoconvertible fluorescence protein that changes from green (Kaede-green) to red (Kaede-red) upon exposure to ultraviolet light (UV)^19,20^. After labeling the skin cells with red fluorescence in this system (Supplementary Fig. 2), we evaluated the number of migrated Kaede-red^+^ cutaneous DCs and γδ T cells in dLNs after IMQ treatment by flow cytometry. RvE1 potently reduced the number of Kaede-red^+^ DCs and Kaede-red^+^ γδ T cells in dLNs (Fig. 3b–e). Consistently, the number of total γδ T cells in dLNs was significantly reduced in RvE1-treated group compared with vehicle-treated group (Supplementary Fig. 3). RvE1 also inhibited the number of Kaede-red^+^ DCs and Kaede-red^+^ γδ T cells in dLNs even in the steady state skin (Fig. 3f,g)^13^.

Because DCs and γδ T cells express BLT1^21,22^, we examined the direct effects of LTB4 and RvE1 on DC and γδ T cell migration, using a transwell migration assay. LTB4 had chemotactic activity on both DCs^13^ and γδ T cells (Fig. 3h,i). These effects were completely cancelled by RvE1 similar to those of the BLT1 antagonist, and again, RvE1 did not enhance the inhibitory effects of the BLT1 antagonist. Collectively, these results suggest that RvE1 may also have anti-psoriatic effects by attenuating DC and γδ T cell migration through inhibition of the LTB4-BLT1 pathway.

### RvE1 regulates the functions of human DCs, Th17 and Tc17 cells

**Figure 4:**
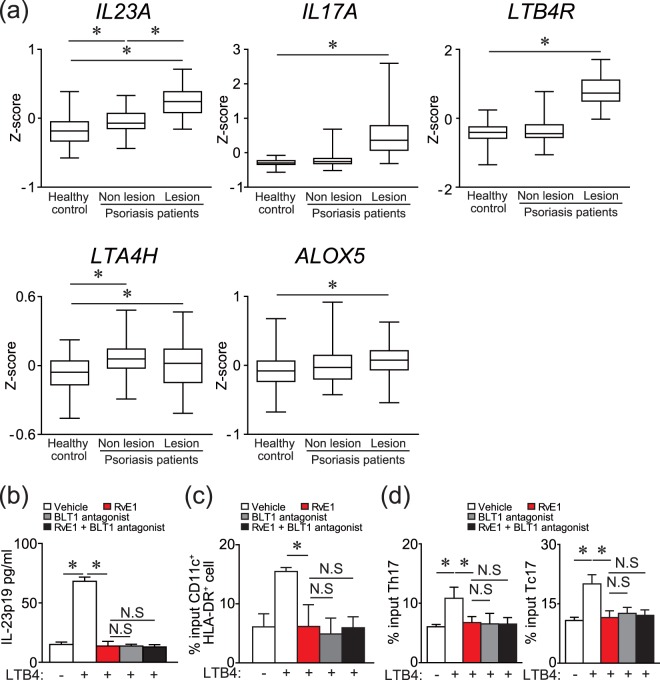
Gene expressions in human psoriatic skin, and inhibitory effects of RvE1 on the IL-23 production by DCs and migration of DCs, Th17 and Tc17 cells. (**a**) Box and whisker plots of mRNA levels of *IL23A*, *IL17A*, *LTB4R*, *LTA4H*, and *ALOX5*. Box and whisker plots of Z scores (*Y*-axis) for skin taken from human healthy controls, and non-lesional and lesional skin taken from psoriasis patients in a public data set (GEO accession no. GSE13355). The length of the box represents the distance between 25% and 75%, and the horizontal line inside the box represents the group median. The whiskers indicate minimum and maximum values. All *p*-values were obtained by nonparameteric Wilcoxon-Mann-Whitney test. (**b**) The effects of LTB4 (100 nM), RvE1 (100 nM) and a BLT1 antagonist (10 nM) on IL-23p19 production by human monocyte-derived DCs. The amount of IL-23p19 in culture supernatant was measured by ELISA. (**c**) Transwell migration assay for human MoDCs, Tc17, and Th17 cells. Thirty minutes after RvE1 (100 nM), a BLT1 antagonist (U-75302) (10 nM), or vehicle treatment, human MoDCs, Tc17 and Th17 cells were tested for transmigration for 5 h (BMDCs) or 3 h (Tc17 and Th17 cells) in the presence of vehicle or LTB4 (10 nM) in the lower (BMDCs) or upper (Tc17 and Th17 cells) chamber. Results are expressed as the mean ± SD. All *p*-values were obtained by Student’s *t-*test: *p < 0.05. N.S indicates no significant difference. Data are representative of at least three independent experiments.

The above results suggest that LTB4 may also be produced in human psoriasis lesions and is involved in its development, and RvE1 may have regulatory roles in it. To ensure the human relevance, we examined the mRNA expressions of *LTA4H*, *ALOX5*, and *LTB4R* (a human LTB4 receptor) in human psoriasis lesions, using a public microarray data set^23^. Consistent with previous studies showing increased LTB4 production in psoriatic lesions^24^, the mRNA expressions of *LTA4H, ALOX5*, and *LTB4R* were marginally but significantly upregulated in the lesional skin compared with healthy control skin, similar to *IL17A* and *IL23A* **(**Fig. 4a**)**.

Thus, we next examined the effects of LTB4 and RvE1 on IL-23p19 production of human DCs, using monocyte-derived DCs (MoDCs). Similar to the results in mouse cells, LTB4 induced IL-23p19 production of human MoDCs. This effect of LTB4 was cancelled by RvE1 and a BLT1 antagonist (Fig. 4b). No additional inhibitory effect of RvE1 was observed in the presence of a BLT1 antagonist.

We further examined the effects of LTB4 and RvE1 on the migration ability of human DCs, Th17 and Tc17 cells. LTB4 showed the chemotactic activity to human DCs (Fig. 4c). On the other hand, although LTB4 did not show significant chemotactic activity to Th17 and Tc17 cells, LTB4 increased the chemokinesis of Th17 and Tc17 cells (Fig. 4d and Supplementary Fig. 4). All these effects by LTB4 were blocked by RvE1 and a BLT1 antagonist (Fig. 4c,d). RvE1 again showed no additional inhibitory effect in the presence of a BLT1 antagonist (Fig. 4c,d). Collectively, these results suggest a possible involvement of LTB4-BLT1 signaling in the development of human psoriasis, which may be regulated by RvE1.

## Discussion

The beneficial impact of omega-3 PUFAs on psoriasis has long been suspected by various epidemiological studies^6^. Recently, it has also been reported that *Fat1*-transgenic mice, which create omega-3 PUFAs from omega-6 PUFAs endogenously by the action of *FAT1*, exhibit reduced psoriatic dermatitis in IMQ-induced psoriasis model^25^, further suggesting the protective roles of omega-3 PUFAs in psoriasis. However, the precise molecular mechanisms by which omega-3 PUFAs regulate the development of psoriasis have remained elusive.

Here, we show the novel mechanisms of antipsoriatic effects of an omega-3 PUFA derivative, RvE1. RvE1 inhibits LTB4-induced IL-23 production by DCs, and also inhibits the migration of DCs and γδ T cells in psoriatic dermatitis in mice. Furthermore, RvE1 suppresses LTB4-induced IL-23 production and migration of human MoDCs and/or Th17/Tc17 cells. Although it remains unclear whether and how much RvE1 is actually produced in the psoriatic lesions, a recent report indicated an imbalance between pro-resolving and pro-inflammatory mediators in the psoriatic skin lesions^26^. Among the pro-resolving mediators, RvD5 was upregulated in the psoriatic lesions, and showed inhibitory effects on the expression of IL-24 and S100A12 from keratinocytes^26^, both of which are suggested to facilitate the development of psoriasis^27^. These studies^26^ including ours suggest possible regulatory mechanisms of omega-3 PUFAs in psoriasis and may propose a potential of omega-3 PUFA-metabolites as therapeutic targets of psoriasis.

It is known that resolvins, including RvE1, exert two different actions in inflammatory conditions: anti-inflammation and pro-resolution^28^. These two actions are not equivalent, because anti-inflammation indicates inhibition of exogenous and endogenous pro-inflammatory mediators, while pro-resolution indicates an active process to terminate the inflammation, such as via clearance of apoptotic cells by macrophages^28^. In this regards, in addition to the anti-inflammatory effects of RvE1 as revealed by our study, RvE1 may also exert pro-resolving roles in psoriatic dermatitis and contribute to the recovery of the inflammatory skin to homeostasis. Since several analogues of RvE1 have already been established^29^, it is of interest to test these analogues as the treatment for inflammatory skin diseases, including psoriasis.

We show that the antipsoriatic effects of RvE1 are mediated through the inhibition of LTB4-BLT1 signaling. Involvement of LTB4 in psoriasis has been suspected in the context of neutrophil infiltration in the skin^30^. For example, exacerbation of psoriasis by non-steroidal anti-inflammatory drugs (NSAIDs) was considered due to increased LTB4 production in the skin by the actions of NSAIDs and a subsequent increase of neutrophil infiltration^31^. Indeed, neutrophils express BLT1, and a recent report shows that BLT1-deficient mice exhibit impaired neutrophil infiltration in the skin and show reduced psoriatic inflammation^30^. Since RvE1 as well as other resolvins regulate the migration of neutrophils in both mice^7,9^ and humans^32^, RvE1 may also exert anti-inflammatory effects on our psoriasis model by regulating migration of neutrophils. Nevertheless, our results have significance in revealing the novel actions of LTB4 on the psoriatic dermatitis: facilitation of IL-23 production by DCs and migration of DCs and γδ T cells, which were inhibited by RvE1.

We and others have previously discovered that cutaneous γδ T cells migrate from skin to dLNs and modulate cutaneous immune responses^18,33^, however, the factors that control the migration have remained unclear. Here, we revealed that RvE1 inhibits γδ T cell migration in the skin, and that LTB4 exerts chemotactic activity to γδ T cells, suggesting that LTB4-BLT1 signaling mediates the cutaneous γδ T cell migration in mouse. Although LTB4 did not exert chemotactic activity to human Th17 and Tc17 cells, it exerted chemokinetic activity to them, which may enhance their responses to chemokines for skin infiltration^34^, and may promote the psoriatic dermatitis in human.

Then, what is the merit of omega-3 PUFAs derivatives as therapeutics for psoriasis? Currently, biologics such as IL-23 inhibitors and IL-17 inhibitors are widely used for the treatment of psoriasis, and have revolutionized the therapeutics for psoriasis. Despite their potent therapeutic effects, however, biologics have several critical disadvantages, one of which is an increased risk of infection due to their immunosuppressive effects^2^. Targeting omega-3 PUFA derivatives may be advantageous in this regard, since the primary functions of omega-3 PUFA-derived anti-inflammatory lipid mediators are to limit the excess inflammation that is deleterious to hosts, and would thus be less likely to cause unnecessary immunosuppressive effects^8^. Development of new treatment options using omega-3 PUFA derivatives may lead to the improved management of psoriasis.

In summary, our findings suggest a novel pathway of omega-3 PUFAs-mediated antipsoriatic effects. Targeting the omega-3 PUFA derivatives can be considered a possible strategy for the new treatment of psoriasis.

## Methods

### Animals and reagents

Female C57BL/6 (B6) mice were purchased from Japan SLC (Hamamatsu, Japan). All experiments were conducted on 8- to 12-week-old mice. Kaede transgenic mice and BLT1-deficient mice on a B6 background were generated as previously described^20,35^. RvE1 was synthesized as previously described^36^, and was stored in −80 °C at the concentration of 1 mg/ml in ethanol, and was prepared at the indicated concentration every time for each experiment. LTB4 and a BLT1 antagonist (U-75302) were purchased from Cayman Chemical (Ann Arbor, MI).

### IMQ-induced psoriasis model

Eight- to eleven-week-old mice received a daily topical dose of 10 mg of IMQ-containing cream (5%) (Mochida Pharmaceutical, Tokyo, Japan) on both ears for seven consecutive days. Vehicle or RvE1 (200 ng/body) was administered intravenously every day 30 min before IMQ application. Ear thickness was measured before and 24 h after each IMQ application using a thickness gauge (Teclok, Nagano, Japan), and the difference was expressed as ear swelling.

### Histology and immunostaining

At day seven after topical IMQ application, ears were excised, fixed in 10% formaldehyde, and embedded in paraffin. Sections of 5 μm thickness were stained with hematoxylin and eosin.

### Flow cytometry

To prepare the skin cell suspensions, the ears were cut and split into dorsal and ventral halves, and the cartilage was removed. The ear skin and lymph nodes (LNs) were incubated for 1 h or 20 min, respectively, in complete RPMI (cRPMI) (RPMI 1640; Sigma-Aldrich, St. Louis, MO) containing 10% heat-inactivated fetal calf serum (Invitrogen, San Diego, CA), 50 μM 2-mercaptoethanol, 2 mM l-glutamine, 25 mM N-2-hydroxyethylpiperazine-N′-2-ethanesulfonic acid, 1 mM nonessential amino acids, 1 mM sodium pyruvate, 100 U/ml penicillin, and 100 μg/ml streptomycin), 1000 unit/ml of collagenase II (Worthington Biochemical, Freehold, NJ), and 0.1 mg/ml of DNaseI (Sigma-Aldrich, St. Louis, MO). The cell suspensions were filtered with 40 μm of cell strainer and stained with the indicated antibodies.

Cells were stained with various combinations of fluorescence-conjugated monoclonal antibodies (mAbs) and analyzed with a Fortessa flow cytometer (BD Biosciences, San Diego, CA) and FlowJo software (TreeStar, San Carlos, CA). For mouse cell analysis, the following antibodies were used: FITC-conjugated anti-Gr1 mAb (eBisocience); PE-Cy7-conjugated anti-CD11c, anti-CD45 (eBioscience) and anti-IL-17A mAbs (eBioscience); and PB-conjugated anti-γδ TCR and anti-MHC classII mAbs (Biolegend, San Diego, CA). All mAbs were used at a concentration of 1 to 5 mg per 10^6^ cells, and each incubation was performed for 30 min at 4 °C, followed by two washes in PBS supplemented with 5% fetal calf serum and 0.02% sodium azide. To exclude dead cells, cells were stained with fixable viability dye eFluor 780 (eBioscience), according to the manufacturer’s instructions. For human cell staining, the following antibodies were used: APC-conjugated anti-CD3 and anti-CD11c mAbs (eBioscience); PerCP-Cy5.5-conjugated anti-CD8 (eBioscience) mAbs; FITC-conjugated anti-IL-17A mAb (Biolegend); and PerCP-Cy5.5-conjugated anti-HLA-DR (BD Biosciences) mAb. For intracellular cytokine staining, cells were incubated in the presence of Goldi Stop (BD Biosciences) for 4 h under stimulation of phorbol myristate acetate (PMA) and ionomycin. Intracytoplasmic cytokines were detected in permeabilized cell suspensions using a BD cytofix/cytoperm Plus Kit (BD Biosciences).

### IL-17A production in γδ T cells

LN cells (2 × 10^6^cell/well in 96well plate) were stimulated with recombinant mouse IL-23 (10 μg/ml) (R&D Systems, Minneapolis, Minnesota) in the presence or absence of RvE1 (100 nM) for 24 h. Golgistop was added in the last 4 h. Intracellular staining for IL-17A in γδTCR^mid+^ cells was performed, and was subjected to a flow cytometric analysis.

### Quantitative real-time polymerase chain reaction (PCR) analysis

Quantitative real-time PCR analysis was performed as reported previously^17^ with some modifications. Briefly, total RNA was extracted from ear skin using a Trizol RNA extraction kit (Invitrogen). cDNA was reverse transcribed from the total RNA samples using a Prime Script RT reagent kit (Takara Bio, Otsu, Japan). Quantitative real-time PCR was performed by monitoring the synthesis of dsDNA during the various PCR cycles using SYBR Green I (Takara Bio) and the 7500 Real-Time PCR System (Applied Biosystems, Foster City, CA) according to the manufacturer’s instructions. All primers were obtained from Greiner Japan (Tokyo, Japan). The primer sequences were *Gapdh*, 5′-AGG TCG GTG TGA ACG GAT TTG-3′ (forward), 5′-GGG GTC GTT GAT GGC AAC A-3′ (reverse); *Il17a*, 5′-CTC CAG AAG GCC CTC AGA CTA C-3′ (forward), 5′-GGG TCT TCA TTG CGG TGG-3′ (reverse); *Il23a*, 5′-AAC TCC TCC AGC CAG AGG ATC A-3′ (forward), 5′-TCT TGG AAC GGA GAA GGG GG-3′ (reverse); *Il12b*, 5′-GGT GTA ACC AGA AAG GTG CG-3′ (forward) and 5′-TAG CGA TCC TGA GCT TGC AC-3′ (reverse); *Tnf*, 5′-TGC CTA TGT CTC AGC CTC TTC-3′ (forward) and 5′-GAG GCC ATT TGG GAA CTT CT-3′ (reverse); *Ccl20*, 5′- GCC TCT CGT ACA TAC AGA CGC-3′ (forward) and 5′-CCA GTT CTG CTT TGG ATC AGC-3′ (reverse); *Alox5*, 5′-GGG CTG TAG CGA GAA GCA TC-3′ (forward) and 5′-CAC GGT GAC ATC GTA GGA GT-3′ (reverse); *Lta4h*, 5′-GAG GTC GCG GAT ACT TGC TC-3′ (forward) and 5′-CTC CTG TGA CTG GAC CGT G-3′ (reverse). For each sample, triplicate test reactions and a control reaction lacking reverse transcriptase were analyzed for expression of the genes, and results were normalized to those of the housekeeping *Gapdh* mRNA.

### Photoconversion

Photoconversion of skin cells was performed as previously described^20^. Briefly, Kaede transgenic mice were anesthetized, their backs were shaved, and they were exposed to violet light at 95 mW/cm^2^ with a 436 nm bandpass filter using spot UV curing equipment for 10 min (SP500; USHIO, Tokyo, Japan). After violet irradiation, the fluorescence of skin cells turns from green (Kaede green) to red (Kaede red).

### Generation of BMDCs

For BMDC generation, 5 × 10^6^ BM cells generated from wild-type (WT) mice were cultured in cRPMI supplemented with 10 ng/mL of recombinant murine granulocyte-macrophage colony-stimulating factor (GM-CSF) (PeproTech, Rocky Hill, NJ) for 5 to 7 days.

### Preparation of MoDCs

Monocytes were isolated by the standard Ficoll-Paque method (Pharmacia, Uppsala, Sweden) from peripheral blood mononuclear cells (PBMCs) obtained from healthy blood donors. CD14^+^ cells purified with auto MACS (Miltenyi Biotec, Auburn, CA, USA) were cultured in cRPMI supplemented with 100 ng/ml recombinant human GM-CSF (R&D Systems, Minneapolis, MN) and 50 ng/ml recombinant human IL-4 (R&D Systems) for 7 days.

### Transwell migration assay

The single cell suspensions from skin, BMDCs, MoDCs and PBMCs were pretreated with RvE1 (100 nM), a BLT1 antagonist (U-75302) (10 nM), or vehicle for 30 min. In the presence of LTB4 (10 nM) or vehicle in the upper or lower chamber of the transwell, these cells were placed in the upper chamber. Migrated cells across uncoated 5 μm transwell filters (Corning Costar, Corning, NY) were analyzed by flow cytometry. RPMI-1640 with 0.5% fatty acid-free bovine serum albumin (Calbiochem, San Diego, CA) was used as the culture medium.

### Enzyme-linked immunosorbent assay (ELISA)

BMDCs or MoDCs were cultured with vehicle, RvE1 (100 nM), a BLT1 antagonist (U-75302) (10 nM), or LTB4 (10 nM) for 24 h. The concentration of IL-23p19 in culture supernatant was measured by ELISA (mouse IL-23 ELISA kit, eBioscience; human IL-23 ELISA kit, Thermo Fisher Scientific, Waltham, MA) according to the manufacturer’s instructions.

### Data analysis of microarray

Expression data for 58 psoriasis cases and 64 healthy controls were obtained from a public data set deposited in the National Center for Biotechnology Information (NCBI) Gene Expression Omnibus (GEO) database (GEO accession no. GSE13355)^23^. Patient and sample information were described previously. Briefly, skin biopsies were obtained from the buttocks of 64 control individuals and 2 biopsies (1 involved, 1 uninvolved) from 58 psoriatic subjects. Isolated RNA from each biopsy were subjected to Affymetrix U133 Plus 2.0 arrays to evaluate gene expressions, and an index Z value of each element was generated. *p*-values were calculated by nonparameteric Wilcoxon-Mann-Whitney test.

### Statistical analysis

All statistical analyses were carried out using GraphPad Prism 4.0 (San Diego, CA). The Student’s *t*-test or nonparameteric Wilcoxon-Mann-Whitney test was used to calculate statistical differences. All *p*-values less than 0.05 were considered statistically significant.

### Study approval

This study was conducted with the approval of, and in accordance with, the Guidelines for Animal Experiments of the Kyoto University Graduate School of Medicine. The human study was approved by the ethics committee of the Kyoto University Graduate School of Medicine. Informed consent was obtained from all subjects according to the declaration of Helsinki.

## Electronic supplementary material

Supplementary Information
