# Supplementary material for: Resolvin E1 attenuates murine psoriatic dermatitis
Source: Sci Rep. 2018 Aug 8;8:11873. doi: 10.1038/s41598-018-30373-1 (PMC6082864; doi:10.1038/s41598-018-30373-1)
Supplement: Supplementary file 1 — Supplementary Information [file 41598_2018_30373_MOESM1_ESM.pdf]

Resolvin E1 attenuates murine psoriatic dermatitis

Yu Sawada MD, PhD [1, 2], Tetsuya Honda MD, PhD [1 *], Satoshi Nakamizo MD, PhD [1], Atsushi Otsuka MD, PhD [1], Narihito Ogawa PhD [3], Yuichi Kobayashi PhD [3], Motonobu Nakamura MD, PhD [2], and Kenji Kabashima MD, PhD [1, 4 *]

1. Department of Dermatology, Kyoto University Graduate School of Medicine, Kyoto, Japan
2. Department of Dermatology, University of Occupational and Environmental Health, Kitakyushu, Japan
3. Department of Biomolecular Engineering, Graduate School of Bioscience and Biotechnology, Tokyo Institute of Technology, Tokyo, Japan
4. Singapore Immunology Network (SIgN) and Institute of Medical Biology, Agency for Science, Technology and Research (A*STAR), Singapore

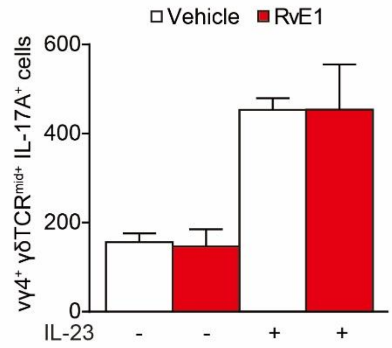

Figure S1. IL-17A production by γδ T cells.
Twenty-four hours after the stimulation with recombinant IL-23 (10µg/ml) in the presence or absence of RvE1 (100 nM), LN suspension cells were stained with anti-γδTCR and anti-IL-17A mAbs, and subjected to a flow cytometric analysis. N.S, no significant difference.

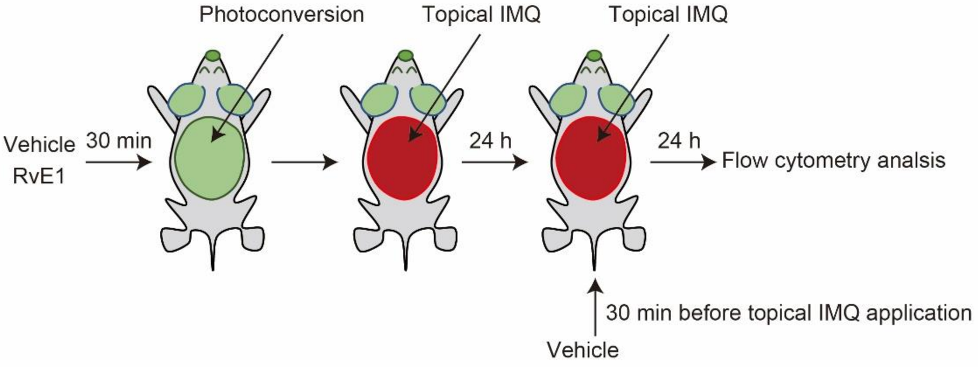

Figure S2. A schema of the photo-labelling system using Kaede transgenic mice.
Thirty minutes after the administration of vehicle or RvE1 (200 ng/mouse), the shaved back skin of Kaede transgenic mice was irradiated with violet light (photoconversion), and treated with IMQ. Twenty-four hours after the photoconversion, vehicle or RvE1 was again administered, and IMQ was applied 30 min later. Forty-eight hours after the photoconversion, the brachial dLNs were collected, stained with anti-CD11c, anti-MHC class II, and anti-γδTCR mAbs, and subjected to flow cytometric analysis.

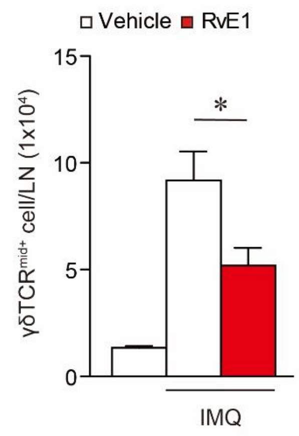

Figure S3. The number of γδTCR+ cells in dLN.

The number of γδTCR^{mid+} cells in the steady state and IMQ treated mice for 7 days with RvE1 or vehicle administration were subjected to the flow cytometry.

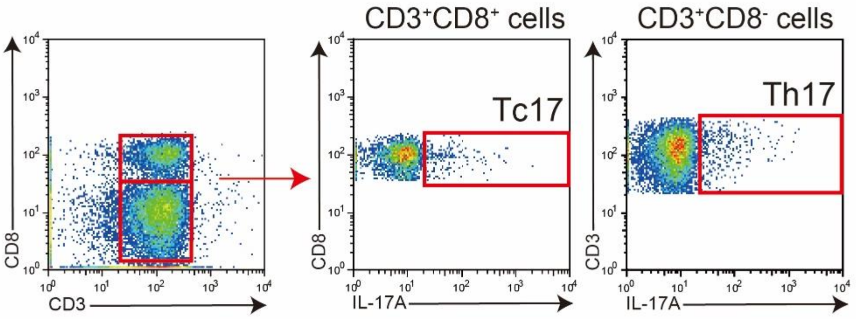

Figure S4. A representative FACS plot of Tc17 and Th17 cells.

PBMCs were stimulated with PMA and ionomycin for 4 hours and Golgistop was added. Then, production of IL-17A by PBMCs was determined at the single-cell level by intracellular cytokine staining. The numbers outside the upper and lower right quadrants represent the percentage of IL-$17A^{+}$ cells in $CD3^{+}CD8^{+}$ or $CD3^{+}CD8^{+}$ cells.
